# MLVA for *Salmonella enterica* subsp. *enterica* Serovar Dublin: Development of a Method Suitable for Inter-Laboratory Surveillance and Application in the Context of a Raw Milk Cheese Outbreak in France in 2012

**DOI:** 10.3389/fmicb.2017.00295

**Published:** 2017-02-27

**Authors:** Marie-Léone Vignaud, Emeline Cherchame, Muriel Marault, Emilie Chaing, Simon Le Hello, Valerie Michel, Nathalie Jourdan-Da Silva, Renaud Lailler, Anne Brisabois, Sabrina Cadel-Six

**Affiliations:** ^1^Université PARIS-EST, Agence Nationale de Sécurité Sanitaire de l’Alimentation, de l’Environnement et du Travail, Laboratory for Food SafetyMaisons-Alfort, France; ^2^French National Reference Center for E. coli, Shigella and Salmonella, Institut PasteurParis, France; ^3^Department of Dairy Products, Center of Expertise for the Food IndustryLa Roche-sur-Foron, France; ^4^French National Public Health AgencySaint-Maurice, France

**Keywords:** *Salmonella* Dublin, MLVA analysis, PFGE analysis, protocol for inter-laboratory surveillance, raw milk cheese, foodborne outbreak

## Abstract

*Salmonella enterica* subspecies *enterica serovar* Dublin (*S*. Dublin) figures among the most frequently isolated *Salmonella* strains in humans in France. This serovar may affect production and animal health mainly in cattle herds with corresponding high economic losses. Given that the current gold standard method, pulsed-field gel electrophoresis (PFGE), provides insufficient discrimination for epidemiological investigations, we propose a standard operating procedure in this study for multiple-locus variable number tandem repeat analysis (MLVA) of *S.* Dublin, suitable for inter-laboratory surveillance. An *in silico* analysis on the genome of *S.* Dublin strains CT_02021853 was performed to identify appropriate microsatellite regions. Of 21 VNTR loci screened, six were selected and 401 epidemiologically unrelated and related strains, isolated from humans, food and animals were analyzed to assess performance criteria such as typeability, discriminatory power and epidemiological concordance. The MLVA scheme developed was applied to an outbreak involving *Saint-Nectaire* cheese for which investigations were conducted in France in 2012, making it possible to discriminate between epidemiologically related strains and sporadic case strains, while PFGE assigned only a single profile. The six loci selected were sequenced on a large set of strains to determine the sequence of the repeated units and flanking regions, and their stability was evaluated *in vivo* through the analysis of the strains investigated from humans, food and the farm environment during the outbreak. The six VNTR selected were found to be stable and the discriminatory power of the MLVA method developed was calculated to be 0.954 compared with that for PFGE, which was only 0.625. Twenty-four reference strains were selected from the 401 examined strains in order to represent most of the allele diversity observed for each locus. This reference set can be used to harmonize MLVA results and allow data exchange between laboratories. This original MLVA protocol could be used easily and routinely for monitoring of serovar Dublin isolates and for conducting outbreak investigations.

## Introduction

*Salmonella enterica* subspecies *enterica* serovar Dublin (*S.* Dublin) is one of the most frequently encountered *Salmonella* in cattle in the European Union. Data from the ANSES *Salmonella* Network (jointly with the National Reference Laboratory) show that this serovar contends with the Typhimurium serovar for the top spot in the ranking of the serovars most frequently isolated in cattle in France. Between 2002 and 2010, *S*. Dublin was outright the most common one (European Food Safety Authority [EFSA], 2015). Infected cattle may develop several clinical signs mainly characterized by i/ diarrhea, pneumonia and death in calves and adult cattle, and ii/ abortion and decreased milk yield in cows (Nielsen et al., 2013).

Beyond the significant economic losses caused in the bovine sector, *S*. Dublin is of concern to public health because it is potentially zoonotic and can cause gastro-intestinal disease and severe infection in humans (O’Leary, 2014). It can be transmitted to humans via meat and dairy products (Maguire et al., 1992). *Salmonella* Dublin outbreaks have occurred regularly in France in the last few years. Protracted difficulties are probably the result of several factors: persistence in the environment, e.g., in wet and dried feces (Findlay, 1972; Plym-Forshell and Ekesbo, 1996), persistence in cattle herds (Clegg et al., 1986; Boqvist and Vagsholm, 2005), a carrier state or prolonged shedding, or reinfection of susceptible animals (Centers for Disease Control [CDC], 1984; Nielsen, 2009). Persistently infected cows can shed the bacteria intermittently in their feces for prolonged periods without ever showing signs of disease, making control of infection particularly difficult at the breeding level (Nielsen et al., 2013).

In France, Dublin varied between the 20th to the 9th position (*n* = 45 to 120 clinical isolates) of the most frequently isolated serovars in humans between 2000 and 2013, with a peak observed in 2012 (Weill and Le Hello, 2013). During the same period, *S.* Dublin contamination in the bovine sector recorded by the *Salmonella* Network increased, with a peak observed in 2013. The relative frequency of *S*. Dublin detected by the *Salmonella* Network in 2010 for cattle and dairy products was 10.4 and 57.6%, respectively, compared to 37.7 and 5.8% for *S*. Typhimurium (Inventaire du Réseau Salmonella, 2010; Lailler et al., 2012). The increased contamination in humans, and in the animal and food sectors, demonstrates the need for a method that can monitor *S.* Dublin strains alongside outbreak investigations. PFGE, considered as the ‘gold standard’ among molecular typing methods, is routinely used for monitoring and surveillance, as well as investigation of outbreaks. Nevertheless, the discriminatory power for *S*. Dublin is low and does not enable investigation and tracking of the source of contamination during foodborne outbreaks (FBOs; Liebana et al., 2002). A study conducted by the *Salmonella* Network on a large panel of Dublin strains highlighted the genetic homogeneity of this serovar (Kerouanton et al., 1996). Moreover, the ANSES *Salmonella* Network’s PFGE database shows that 84% of the *S.* Dublin strains collected since 2003 were assigned to the same profile (SDUBXB0003 for 153/183 strains).

Several methods have already been proposed in the past as an alternative to PFGE, including multilocus enzyme electrophoresis (MLEE; Beltran et al., 1988), ribotyping (Chowdry et al., 1993), restriction fragment length polymorphism analysis (Kerouanton et al., 1996), restriction enzyme fragmentation pattern (REFP) analysis (Olsen and Skov, 1994), various PCR techniques, IS200 typing, and quantitative evaluation of fatty acid methyl esters (FAME; McDonough et al., 1999). Nevertheless, among all these methods, either the discriminatory power was insufficient or the process was laborious, time-consuming and expensive. More recently, MLVA was proposed for subtyping *Salmonella* subsp. *enterica* strains (Ramisse et al., 2004). This method was shown to have better performance than historical methods by displaying higher discriminatory power for *Salmonella* serovars such as Typhimurium and Enteritidis (Lindstedt et al., 2004; Hopkins et al., 2011). Moreover, this method proved very useful in investigating FBOs and facilitated the analysis since it requires no specific technical expertise (Wattiau et al., 2011 review).

In this study, we developed a new MLVA protocol for high discriminatory typing of *S.* Dublin in accordance with the guidelines published by Nadon et al. (2013) on the development and application of MLVA methods as tools for inter-laboratory surveillance. A reference set of strains and a scheme for harmonization of results are also proposed to allow data exchange between laboratories. In order to assess the discriminatory power of the MLVA protocol developed here and the stability of tandem repeats (TRs), we used a set of 401 strains isolated between 1929 and 2015 from three different collections (*Salmonella* Network, National Reference Centre for *Salmonella* and Centre of Expertise for the Food Industry). In particular, this panel of strains comprised human and food isolates recovered in the framework of a FBO investigation that occurred in 2012 in France.

In August 2012, the French National Public Health Agency (SpFrance) detected an unusual increase in cases of human *S.* Dublin infection. Epidemiological investigations highlighted an association between cases and the consumption of raw milk cheese (Saint-Nectaire). Two producers of cheese were identified as potential sources of contamination. Enhanced self-monitoring on the two farms was implemented and further epidemiological and microbiological investigations were conducted. Fifteen FBOs were recorded corresponding to more than 100 cases due to *S*. Dublin (Giron et al., 2013). On 4 September 2012, a withdrawal/recall of contaminated batches of Saint-Nectaire cheese was ordered by the General Directorate for Food (regional office) and of the 7580 kg of cheese produced by the two farms, 1335 kg (17%) were immediately withdrawn from sale.

*Salmonella* Dublin strains isolated from the two different cheese producers suspected to be implicated in this FBO and the clinical strains from the patients were analyzed with the MLVA protocol proposed in this study. Since 2012, this MLVA Dublin protocol is used routinely in our laboratory in order to analyze strains of *S*. Dublin for monitoring and surveillance, investigation of outbreaks, and official controls. Indeed, there is still concern in this regard because new cases of *S*. Dublin associated with raw milk cheeses were reported over the summer months of 2016.

## Materials and Methods

### Bacterial Strains

A total of 401 *S.* Dublin strains were used for the development of the MLVA scheme. Among them, 109 strains were isolated from animals, 198 from food products and 94 from humans (see **Supplementary Table S1**). The strains isolated from animals and food sources were collected by the *Salmonella* Network of ANSES and the Centre of Expertise for the Food Industry (Actalia); the clinical strains came from the National Reference Centre for *Salmonella* at Institut Pasteur in Paris. This panel included 251 epidemiologically unrelated and 150 epidemiologically related strains isolated along the cheese production chain. Among these, 13 strains were recovered from the Saint-Nectaire samples during the 2012 outbreak investigations. All strains were identified as belonging to serovar *Salmonella* Dublin according to the White-Kauffmann-Le Minor scheme (Grimont and Weill, 2007).

### Pulsed-Field Gel Electrophoresis

Within the panel of 401 strains used for the development of the MLVA scheme, 51 *Salmonella* Dublin strains, isolated from 2002 to 2011, were PFGE subtyped according to a standardized protocol (PulseNet, 2013, EU) with some modifications in the composition of the cell lysis buffer and the concentration of the enzyme *Xba*I (Sigma–Aldrich, France). The cell lysis buffer was 1M Tris: 250 mM EDTA, pH 8.0 + 10% sarkosyl, and the restriction enzyme was five times less concentrated. This analysis of PFGE patterns was performed using BioNumerics^®^ software v.6.6 (Applied Maths, Belgium) and comparison of patterns was carried out by building a dendrogram (Dice coefficients, the UPGMA method and position tolerance set at 1%). Each strain profile was assigned to a PFGE pattern corresponding to a unique pattern. Designation of each PFGE profile was done using a unique nomenclature, e.g., SDUBXB0001.

### Procedure for the Multiple-Locus Variable Number Tandem Repeat Analysis

#### DNA Extraction

Strains were cultured overnight at 37°C on tryptone soya yeast extract agar plate. The extraction was performed with Instagene Matrix (Biorad, France) according to the manufacturer’s instructions for Gram-negative organisms. The clarified supernatant was stored at -20°C. The DNA concentration was measured with Nanodrop ND-1000 (Labtech, France). The DNA concentration of the samples was normalized at a final concentration of 50 ng/μL.

#### Variable Number Tandem Repeat (VNTR) Selection

Twenty-one VNTR markers published from 2003 to 2009 (Kruy et al., 2011, review) to discriminate *S. enterica* subspecies were selected (see **Supplementary Table S2**) and blasted on the genomic sequence of *S.* Dublin strains CT_02021853 (accession No. NC_011205.1/CP001144.1) obtained from http://www.ncbi.nlm.nih.gov/genome/. The presence of TRs was verified using free access TRs Finder software by Benson (1999). The *in silico* analysis performed on the genome of *S.* Dublin strains CT_02021853 revealed that eight loci, on the 21 searched, presented microsatellite sequences. Among these eight loci, six were tested by PCR on 51 *S*. Dublin genomes from the *Salmonella* Network collection to check for the presence of microsatellites and variability of RUs. Finally, all six VNTRs were selected for the development of the MLVA procedure (**Table 1**). The primers for 2 of these VNTRs, STTR3, and SE-2 (developed for Typhimurium and Enteritidis, respectively), were adjusted to the sequence of the genome of *S*. Dublin strains CT_02021853.

**Table 1:** Variable number tandem repeats and primers selected for the MLVA *S*. Dublin scheme.

Locus	Dye	Primers (5′–3′)^a^	Sequence length (bp)^b^	Offset^c^ (bp)	RU^d^ (bp)	RU sequence (bp)
STTR3	PET	CCCCCTA**C**GCCCGATAATGG	409	66	33	GCGGCGATGACAATGTGATCCCGCCCGACGATA
		TGACGCCGTTGCTGAAGGTAATAA				

STTR5	VIC	ATGGCGAGGCGAGCAGCAGT	274	179	6	CACGAC
		GGTCAGGCCGAATAGCAGGAT				

STTR7	VIC	CGCGCAGCCGTTCTCACT	477	265	39	GTAGCGCCGCAGCCGCAGTATCAGCAGCCGCA GCAACCG
		TGTTCCAGCGCAAAGGTATCTA				

SE-2	PET	CTT**AC**GATTATACCTGGATTG	201	168	7	GATGCCG
		TGGACGGAGGCGATAG				

SENTR1	VIC	GCAACAGCAGCAGCAACAG	440	85	45	GAAGCGGCGAAAGCGGCGGCGGACGCGAAGA AGAAAGCGGAAGCC
		CCGAGCTGAGATCGCCAAG				

SENTR3	NED	CTAAACAAGCCGCTCATCCG	493	80	93	CGACCCGAGTAAAGCTGCAGTGGAGGCAGCCA TCGCCCGCGCCAAAGCCCGTAAGCAGGAGCAG CAGGCCGGAAGCGAACCGGTCGAAGCGGT
		ACAACCTGCTGCTGTGCTG				

^a^*the primers presenting underlined letters were designed in this study, the other primers refer to Kruy et al. (2011; review)*.

^b^*sequence length in genome of *S*. Dublin strains CT_02021853 (accession No. NC_011205.1/CP001144.1) obtained from http://www.ncbi.nlm.nih.gov/genome/*.

^c^*length of 5′ and 3′ TR flanking region*.

^d^*Repeated Unit*.

#### PCR Multiplex Amplification

The six selected VNTR regions were targeted in two multiplex assays: M1 (STTR5, STTR7, STTR3) and M2 (SENTR1, SENTR3, SE-2), using the Qiagen multiplex Kit (Qiagen, Germany). The primers were pooled in two premixes, one for M1 the other for M2. The concentrations for each primer were as follow: STTR5 and STTR7: 1.5 μM, STTR3: 3 μM, SENTR1: 2.5 μM, SENTR3: 10 μM and SE-2: 7 μM. M1 and M2 were carried out with a final volume of 25 μL and 15 μL, respectively. Per reaction: 0.9 μL of pre-mix primer for M1, 1.85 μL for M2, and 2 μL of DNA were added. Multiplex PCRs were run on a Verity^®^ thermocycler (Applied Biosystems, France) with different and specific cycling conditions; for M1 reactions:15 min at 95°C, then 25 cycles of 30 s at 95°C, 90 s at 60°C, 90 s at 72°C and ending with a hold at 72°C for 10 min; for M2 reactions: 15 min at 95°C, then 28 cycles of 30 s at 95°C, 90 s at 55°C, 90 s at 72°C and ending with a hold at 72°C for 10 min.

The M1 PCR solution was then diluted to 1/20 and the M2 PCR solution to 1/30 with RNase-free, molecular biology-grade water. Finally, 1 μL of each solution was pooled with 1 μL of 600 Liz internal size marker (Applied Biosystems, France) and 13 μL of formamide for M1 and 0.5 μL of 1200 Liz internal size marker (Applied Biosystems, France) and 10.5 μL of formamide for M2.

The samples were denatured 5 min to 95°C and cooled on ice before being subjected to capillary electrophoresis.

#### Capillary Electrophoresis and Data Analysis

The analyses were carried out on an AB3500 capillary electrophoresis system (Applied Biosystems, France) spectrally calibrated to run filter set G5. The instrument was prepared according to the procedures specified by Applied Biosystems. The standard fragment analysis protocol proposed by the manufacturer was used and positive and negative control isolates were included with each run to follow the drift in results due to the use of the instrument over long time periods. Data were automatically saved as .fsa files and imported into GeneMapper software (Applied Biosystems, France), where each fragment was identified according to color and size. The measured lengths attributed to each peak by GeneMapper were transferred to a .txt file to be normalized with the free access MLVA_Normalizer software^1^, following the instructions of the author (Bachelerie et al., 2016). Then, the MLVA profiles were imported into BioNumerics software version 7.1 (Applied Maths, Belgium) as categorical data. A standard minimum-spanning tree (MST) was generated using the single and double locus variance priority rules, allowed to define the clonality and distance between strains.

The discriminatory power of the single VNTR and of the MLVA method compared to that of PFGE was calculated by Simpson’s index of diversity (DI) according to the formula as described by Hunter and Gaston (1988).

#### Sequencing and Standardization Strains

For sequencing of the VNTR loci, genomic DNA was amplified in a simplex PCR with the same primer sequences used for VNTR detection but with unlabeled forward primers. Sequencing (Life Technologies, Germany) was performed in both directions using both the forward and the reverse primers for all loci to determine the sequence of the TRs and flanking regions. Twenty-four strains with different confirmed numbers of repeats at all loci were chosen as the reference strain panel (**Table 2**).

**Table 2:** Reference strain panel of *Salmonella* Dublin (*n* = 24).

	Reference	MLVA fragment sizes^a^ STTR5, STTR7, STTR3, SENTR1, SENTR3, SE-2	MLVA profile^b^
1	03EB8994SAL	229-594-409-440-494-243	8-8-10-8-4-11
2	10CEB141SAL	235-316-409-395-588-228	9-1-10-7-5-8
3	2014LSAL02934	313-594-409-395-588-187	22-8-10-7-5-3
4	2015LSAL00352	223-594-409-440-493-215	7-8-10-8-4-7
5	2015LSAL00380	325-594-409-395-588-194	24-8-10-7-5-4
6	02EB9210SAL	271-594-409-395-588-194	15-8-10-7-5-4
7	03EB3784SAL	239-594-343-440-588-194	10-8-8-8-5-4
8	09CEB6631SAL	289-594-409-395-588-194	18-8-10-7-5-4
9	10CEB3063SAL	223-316-409-395-588-222	7-1-10-7-5-8
10	10CEB472SAL	247-594-409-395-588-208	11-8-10-7-5-6
11	10CEB80SAL	277-594-409-395-399-194	16-8-10-7-3-4
12	10CEB8244SAL	277-594-409-395-588-194	16-8-10-7-5-4
13	10CEB8798SAL	295-594-409-395-588-187	19-8-10-7-5-3
14	11CEB386SAL	229-594-409-395-588-194	8-8-10-7-5-4
15	11CEB6136SAL	259-594-409-395-588-194	13-8-10-7-5-4
16	11CEB65SAL	217-594-409-440-494-243	6-8-10-8-4-11
17	11CEB6804SAL	301-594-409-395-587-201	20-8-10-7-5-5
18	11CEB6847SAL	289-594-409-395-588-194	18-8-10-7-5-4
19	11CEB6876SAL	295-594-409-395-588-187	19-8-10-7-5-3
20	12CEB3654SAL	265-594-409-395-588-194	14-8-10-7-5-4
21	10CEB2371SAL	229-316-409-395-588-208	8-1-10-7-5-6
22	04_4663	253-478-409-440-493-215	12-5-10-8-4-7
23	201501982	235-316-409-395-588-236	9-1-10-7-5-10
24	201500719	271-478-409-440-493-201	15-5-10-8-4-5

*Isolate Nos. 1–21 were from the *Salmonella* Network (ANSES) and isolate Nos. 22–24 were from the National Reference Centre (*Institut Pasteur*)*.

^a^*The fragment sizes are the true size according to sequence results*.

^b^*The MLVA profile is based on the number of repeated units as described in Nadon et al. (2013). For the incomplete repeats, the copy number is rounded down to the nearest complete copy number*.

#### Reproducibility, Metrology, and Quality Safety Control

Distinct experimental trials were conducted including two different operators and different days of handling (from 2 to 5 days). All instruments were calibrated and metrologically controlled according to ISO NF 17025. The handling was performed under quality safety requirements.

## Results

### Characterization, Diversity, and Allele Distribution of six VNTR Loci

Among the 21 loci included in this study, 8 displayed TRs in the genome of *S.* Dublin strains CT_02021853 by *in silico* analysis. Among these eight loci, two were discarded, STTR4 and SE-6. STTR4 was discarded because the length of the PCR amplicon exceeded the range of detection of the AB3500 capillary electrophoresis system (length of PCR amplicon >1200 bp). SE-6 corresponded to the locus STTR3 even though different authors have given different names to these loci (Kruy et al., 2011, review). For the development of the MLVA protocol, the STTR3 locus, initially described by Lindstedt et al. (2003) was chosen.

Finally, the six selected VNTRs (**Table 1**) enabled identification of 75 different MLVA profiles for the overall panel of 401 strains tested in this study. The most common MLVA profile (19-9-10-7-5-3) was observed for 29% of all strains. The STTR5 and SE-2 loci showed the highest Simpson’s diversity index (DI), 0.805 and 0.625, respectively, with the highest number of different TR alleles (18 and 14 alleles). The lowest Simpson’s diversity values were observed for the STTR3 locus (DI 0.040) with four alleles (**Table 3**). The fraction of strains that have the most frequent allele was calculated by the max(pi) value (range 0.0–1.0). For the STTR5 locus, 39% of the analyzed strains possessed the most common allele (**Table 3**). This result is an additional indicator of the diversity shown by the alleles’ frequencies within the loci.

**Table 3:** Variability of selected VNTRs in 401 strains of *Salmonella enterica* serovar Dublin.

Locus	Simpson’s diversity index	No. of alleles^∗^	max (pi)
STTR5	0.805	17	0.390
STTR7	0.110	5	0.943
STTR3	0.014	2	0.989
SENTR1	0.185	3	0.898
SENTR3	0.195	3	0.895
SE-2	0.625	10	0.435

*^∗^Number of different TRs present at the locus*.

### Reference Strains for MLVA of *Salmonella enterica* Serovar Dublin

**FIGURE 1:**
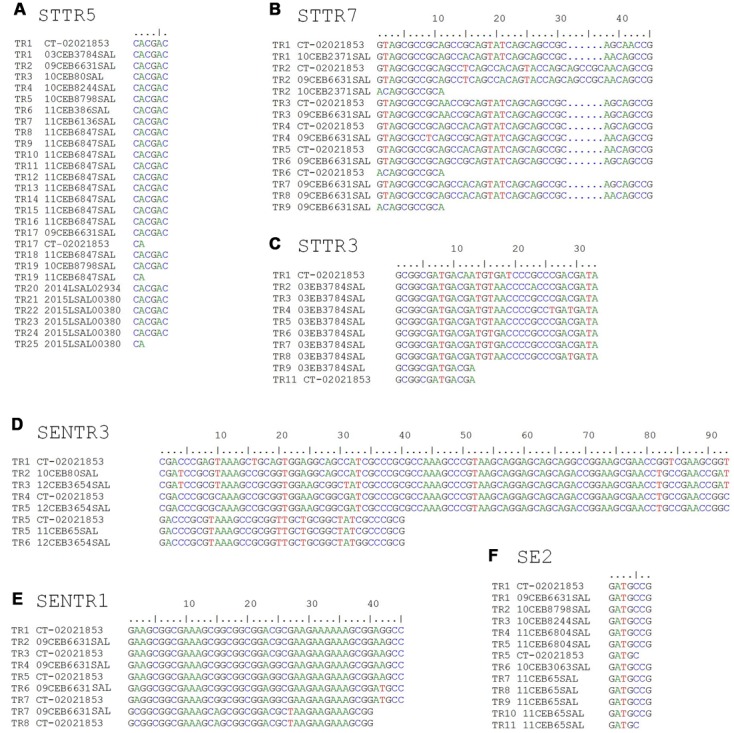
**Alignment of the TR units at the six loci for some reference stains compared with the genome of *S.* Dublin strains CT_02021853. (A)** Locus STTR5; **(B)** locus STTR7; **(C)** locus STTR3; **(D)** locus SENTR3; **(E)** locus SENTR1; **(F)** locus SE-2.

Twenty-four reference strains were selected from the 401 examined strains in order to represent most of the allele diversity observed for each locus. The amplicons from each locus were sequenced (**Table 2**). Data on the 24 reference strains are shown in **Supplementary Table S3**. This table, once compiled with the raw data, can be used as input file for using the MLVA workflow for normalizing MLVA results cited above (Bachelerie et al., 2016). Sequencing confirmed the number of TRs and alignments revealed that the six loci (STTR5, STTR7, STTR3, SENTR3, SENTR1, and SE-2) exhibit no variation in the sequence of the TR unit within a strain and between strains. Single nucleotide polymorphisms (SNPs) were identified in the TRs of the STTR7, STTR3, SENTR3, and SENTR1 loci for 12CEB3654SAL, 09CEB6631SAL and 03EB3784SAL reference strains compared with the genome of *S.* Dublin strains CT_02021853 (**Figure 1**). The TR units at the six loci for the 24 reference strains were aligned. The alignment showed an incomplete repeat for a few strains (**Figure 1**). The number of alleles of such strains was rounded down to the nearest complete copy number, in accordance with the guidelines published by Nadon et al. (2013).

### Discriminatory Power of PFGE and MLVA

Finally, the discriminatory power of the MLVA method developed was compared with that of the PFGE method for the 51 strains for which both methods were performed. PFGE and MLVA profiles are listed in **Supplementary Table S4**. PFGE analysis with *Xba*I sorted the strains into 13 different patterns displaying a discriminatory power with a value of 0.625. MLVA analysis provided 27 different MLVA profiles with a discriminatory value of 0.954. No linear correspondence between the two methods was observed, meaning that MLVA profiles can match, or do not match with a PFGE pattern. The most frequent PFGE pattern (SDUBXB0003) comprised strains characterized by 18 different MLVA profiles.

### Minimum-Spanning Tree Analysis

**FIGURE 2:**
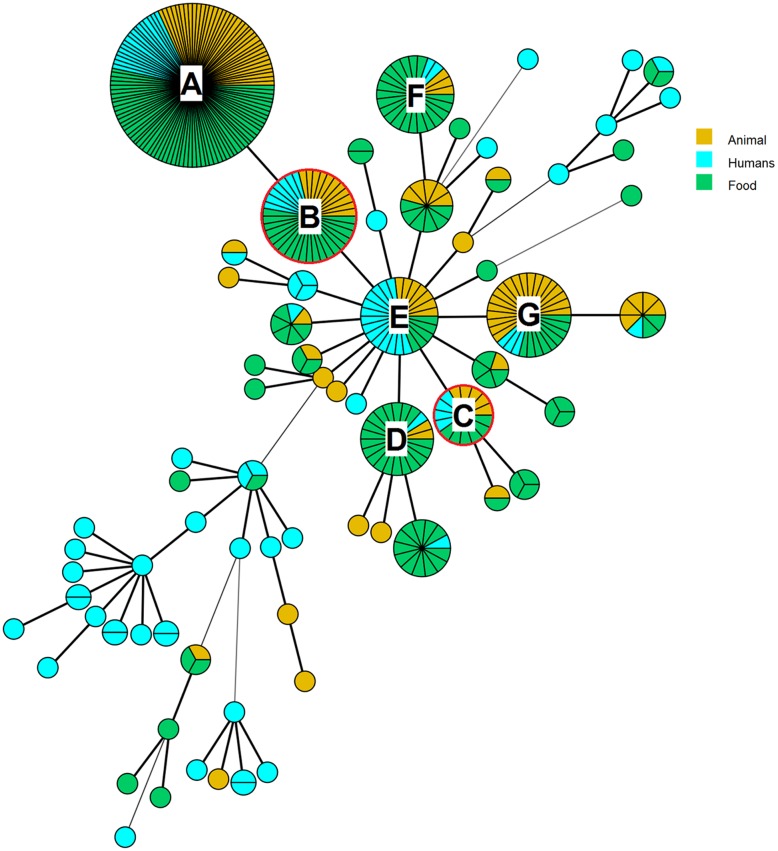
**Minimum-spanning tree on the basis of 6-loci MLVA profiles of 401 *S*. Dublin strains from 1929 to 2015**. The main group (A) is characterized by the MLVA profile 19-8-10-7-5-3. This group includes human strains isolated in 2011, 2012, and 2015 but not related to the Saint-Nectaire outbreak described. The groups (B and C) surrounded in red include strains from the Saint-Nectaire outbreak that occurred in summer 2012 in France. The group (B) is characterized by the MLVA profile 19-8-10-7-5-4. This group includes also human strains isolated in 2015 during an outbreak which source confirmation was not possible. The group (C) is characterized by the MLVA profile 14-8-10-7-5-4. The group (D) is characterized by the MLVA profile 16-8-10-7-5-4, the group (E) by the profile 17-8-10-7-5-4, the group (F) by the profile 15-8-10-7-5-3 and the group (G) by the profile 18-8-10-7-5-4.

A MST was set up from MLVA profiles of the 401 strains according to the human, animal and food sources, and the context of isolation (FBO; see **Supplementary Table S1** and **Figure 2**). The MST displayed a high degree of polymorphism of strains (**Figure 2**). A total of 71 MST groups were observed and 44 of them were represented by a single strain. The main MST group was characterized by the MLVA profile 19-8-10-7-5-3 (**Figure 2**, group A). This profile included 115 strains isolated from 2010 to 2015 from humans, food and animals. This group also includes strains related to an FBO that occurred in 2015. A type of raw milk cheese was suspected to be the source of this FBO, but in the end, no confirmation of the source was possible. Strains isolated during the FBO that occurred in 2012 (**Figure 2**, groups B and C) showed no epidemiological links with those observed in 2015. The six most represented MST groups, after the main one (**Figure 2**, groups B–G), were characterized by 22–38 strains. Human strains are dispersed among each MST group. Among the 27 groups including two or more strains, 54% comprised exclusively human strains. The 94 human, 109 animal, and 198 food strains were grouped into 44, 25, and 33 MLVA profiles, respectively.

### Saint-Nectaire Isolate Analysis from the FBO that Occurred in 2012 and *In vivo* Stability of the VNTRs Selected

The MLVA method was retrospectively applied on a set of food and human strains suspected of being related in the framework of a Saint-Nectaire outbreak in 2012. The *S*. Dublin strains isolated from two different cheese producers in France were differentiated in two distinct MLVA profiles (19-8-10-7-5-4 and 14-8-10-7-5-4; **Figure 2**, groups B and C, respectively). These profiles were those identified for the human strains, leading us to suspect that two different clones were implicated in this FBO (**Table 4**). The MLVA enabled us to discriminate epidemiologically related strains from sporadic case strains, while PFGE assigned only one PFGE profile (SDUBXB0003) for all the FBO and sporadic case strains, and was therefore not discriminant. Two strains from Saint-Nectaire cheese of producer 2 and sampled in the remainder of the meal consumed by patients 3 and 4 (each one from a different district) displayed the same MLVA profiles (14-8-10-7-5-4) as the strains from patients 3 and 4. Two strains recovered from a filter for milk and cheese directly from producer 1 and two strains sampled in the remainder of the meal of patients 1 and 2 (from two different districts) displayed the same MLVA profile 19-8-10-7-5-4. These two MLVA profiles (14-8-10-7-5-4 and 19-8-10-7-5-4) differed for five RUs of the TR STTR5.

**Table 4:** Multiple-locus variable number tandem repeat analysis profiles of FBO strains assigned to the same PFGE profile (SDUBXB0003).

Origin	MLVA profile	Producer	Strain
Ripened Saint-Nectaire	19 – 8 – 10 – 7 – 5 – 4	1	12CEB3653SAL
Ripened Saint-Nectaire	19 – 8 – 10 – 7 – 5 – 4		12CEB3771SAL
Non-ripened Saint-Nectaire	19 – 8 – 10 – 7 – 5 – 4		12CEB3592SAL
Filtered milk	19 – 8 – 10 – 7 – 5 – 4		12CEB3537SAL
			
Patient 1	19 – 8 – 10 – 7 – 5 – 4		201206592
Patient 2	19 – 8 – 10 – 7 – 5 – 4		201206547
			
Ripened Saint-Nectaire	14 – 8 – 10 – 7 – 5 – 4	2	12CEB3654SAL
Ripened Saint-Nectaire	14 – 8 – 10 – 7 – 5 – 4		12CEB3657SAL
			
Patient 3	14 – 8 – 10 – 7 – 5 – 4		201207167
Patient 4	14 – 8 – 10 – 7 – 5 – 4		201207452

## Discussion

Sensitive and specific molecular epidemiological tools are needed to identify the transmission route when FBO events are investigated. Moreover, given the multinational distribution of some food products, collaboration between countries can be crucial in identifying cases and in tracing the source of infection. In this study, we developed an MLVA scheme with 6-loci MLVA to subtype *Salmonella* Dublin strains. This scheme was developed following the guidelines published by Nadon et al. (2013). Twenty-four reference strains were characterized in depth and a scheme for normalization of results was proposed. The discriminatory power of this MLVA scheme was higher than that of the gold standard PFGE method. The 51 strains from the ANSES *Salmonella* Network Collection studied to determine the polymorphism of the 6 *loci* selected for the development of the Dublin MLVA analysis were clustered in 27 different MLVA profiles. PFGE was able to discriminate the same panel of strains in only 13 PFGE profiles displaying a low level of discriminatory power (MLVA DI 0.954 and PFGE DI 0.625, respectively). Previous studies have already shown the higher subtyping sensitivity of MLVA for some *Salmonella* serovars such as *S.* Typhimurium and *S*. Enteritidis, compared to historical methods (Wattiau et al., 2011 review). The MLVA method developed in this study, provided sufficient allelic variation to subdivide the 401 human, animal and food *S*. Dublin strains from France into 71 MLVA profiles. The STTR5 and SE-2 loci had the highest number of alleles and genetic diversity values in agreement with the results of Kjeldsen et al. (2014). These authors reported a four locus MLVA protocol in 2014 with three genomic loci SE-2, SE-5 (equal to the STTR5 locus) and SE-1, plus one locus, SD1, present in the plasmid pCT02021853_74. They also tested five others loci, among which SE-6, that were ultimately not selected because of their low discriminatory power within the panel of 272 strains analyzed. In contrast, we retained the SE-6 locus, called in our study STTR3, because it showed higher discriminatory power within the panel of French strains analyzed. A specificity of higher allelic mutation for the French strains compared to the Danish one for this locus cannot be excluded. Therefore, we did not select loci present on plasmids because of the high variability of the presence or absence of plasmids in *Salmonella* and the ability to acquire or lose such plasmids (Rychlik et al., 2006 review). Nevertheless, we looked for the plasmid-located SD1 locus described by Kjeldsen et al. (2014) and it was in fact not present in any of the studied strains. We also investigated the STTR10pl repeat located on the pSLT plasmid (Lindstedt et al., 2004) within the panel of 401 strains and it was found only in 19 strains (4%).

The MLVA scheme was then used to re-investigate an FBO that occurred in France in 2012 and it was shown to successfully cluster strains from an epidemiologically confirmed outbreak. The *in vivo* stability of the sequences of the RUs was investigated through the isolates analyzed for this FBO. The six *loci* exhibit *in vivo* stability, although the strains differ by sources, time period of sampling and geographical origin. The higher polymorphism identified for the human strains allowed us to distinguish the epidemiologically related strains from other strains isolated among sporadic cases.

Sequencing confirmed the number of TRs. Alignments of sequences revealed that the six loci do not exhibit variation in the flanking sequences and in the sequence of the TR unit within a strain and between strains, even though some SNPs were identified. The SNPs compared with the genome of *S.* Dublin strains CT_02021853 were in the TRs of the STTR3, STTR7, SENTR1, and SENTR3 loci. Sequencing also showed that no insertions and deletions were present in repeat units, except for the STTR7 VNTR for which an insertion of six bases was observed in the reference strain 09CEB6631SAL and in the genome of *S*. Dublin strains CT_02021853.

Given the importance of normalizing the raw results for the comparison of MLVA profiles between laboratories, in this study we defined a set of 24 reference strains and therefore recommend using them for better comparability of results (**Table 2**). This set of reference strains is available from the collection at the ANSES *Salmonella* Network and National Reference Centre of Institut Pasteur. For some serovars, such as Typhimurium (Larson et al., 2009) and Enteritidis (Hopkins et al., 2011), this reference strain set has already been published with the name of the reference strains, the correct MLVA profile, and the true length of each VNTR locus analyzed. The MLVA_normalizer workflow (Bachelerie et al., 2016) enables correction of the raw data obtained with the Dublin MLVA protocol proposed here. The conversion table described herein to ensure compatibility of *S.* Dublin MLVA data between laboratories is also available in **Supplementary Table S3**.

The 6-loci MLVA exhibited high discriminatory power for the 401 strains analyzed. Nine of the MST groups were represented both by human, animal and food strains. The rate of VNTR variation among human strains was higher than that among animal and food strains. We identified 44 different MST groups among the human strains (*n* = 94), 33 among the food strains (*n* = 198), and 25 among the animal strains (*n* = 109). The higher variability of MLVA profiles observed for the human strains could be explained by the longer period analyzed, from 1929 to 2015 for human strains, and from 1972 to 2015 for animal and food strains. The most frequently encountered MLVA profile (19-8-10-7-5-3) included 116 strains that were recovered from cattle (*n* = 37), milk and cheese (*n* = 53) and clinical strains (*n* = 17). When analyzing the presence of strains in animals (mainly cattle) and in humans in our panel of strains from France, we observed substantial overlap of MLVA profiles between these strains. This could indicate that the same strains were responsible for animal and human cases in France. France’s cheese manufacturing sector using cow’s milk (including pressed cheeses and uncooked cheeses) produced 2,50,000 tons in 2012, and grew by 6% in 2013. This segment represents 11% of the total cheese production sector in the country and includes cheeses under protected designation of origin (PDO) or protected geographical indication (PGI). In France, cheese consumption *per capita* is high and relatively stable in the long term. In total, including purchases by households, industry and the consumer via catering, cheese consumption is estimated at more than 26 kg *per capita* per year (Ministere de l’Agriculture, de l’Agroalimentaire, et de la Forêt, 2014). Because of the importance of milk product consumption and production in France, and taking into account recent FBO events, it appears essential to have a typing method that discriminates *S.* Dublin strains for purposes of monitoring and surveillance, investigation of outbreaks, in the frame of in-house and official controls.

## Conclusion

The ANSES Laboratory for Food Safety has been using this MLVA scheme to subtype *S*. Dublin since 2012. The results of this study are in complete agreement with the work presented by Kjeldsen et al. (2014), indicating that MLVA is a beneficial tool for investigating *Salmonella* Dublin in different epidemiological situations.

## Availability of Data and Material

The reference strains for Dublin MLVA typing are from the *Salmonella* Network Collection of ANSES and from the National Reference Centre (Institut Pasteur). They are available by writing to: Réseau *Salmonella*, Laboratoire de Sécurité des Aliments, ANSES; 14 rue Pierre et Marie Curie; 94701 Maisons-Alfort or by emailing: sabrina.cadelsix@anses.fr.

## Author Contributions

SC-S designed, developed, and piloted the experiments for the MLVA scheme. ECha performed experiments during MLVA scheme development. M-LV performed experiments during the development of the MLVA scheme, and analyzed and interpreted the data for the work. MM performed PFGE analyses. VM and SLH provided strains. EChe performed the MST analysis. NJDS, SLH, RL, SC-S, and M-LV were involved in collecting FBO data, strains and analyses. SC-S and M-LV drafted the manuscript. NJDS, RL, SLH, and AB participated in the discussion and reviewed the report. All authors read, commented, and approved the final manuscript.

## Conflict of Interest Statement

The authors declare that the research was conducted in the absence of any commercial or financial relationships that could be construed as a potential conflict of interest.

## Footnotes

1 https://github.com/afelten-Anses/MLVA\_normalizer

## Supplementary Material

TABLE S1 **List of strains analyzed in the present study**.

TABLE S2 **Twenty-one VNTR markers tested**. The 21 VNTR markers published from 2003 to 2009 and collected in the review of Kruy et al. (2011) selected for the study and blasted on the genomic sequence of *Salmonella* Dublin strains CT_02021853 (accession No. NC_011205.1/CP001144.1).

TABLE S3 **Multiple-locus variable number tandem repeat analysis and PFGE profiles for 51 strains used for calculating the discriminatory power of the two typing methods**.

TABLE S4 **Input file with data on the 24 standardization strains of *Salmonella* Dublin**. This table can be used within an MLVA_normalizer workflow for normalizing MLVA results (Bachelerie et al., 2016).

## Abbreviations

DI: discriminatory index
MLVA: multiple-locus variable number tandem repeat analysis
PFGE: pulsed-field gel electrophoresis
RU: repeated unit
VNTR: variable number tandem-repeat
